# Plant Extracts as Skin Care and Therapeutic Agents

**DOI:** 10.3390/ijms242015444

**Published:** 2023-10-22

**Authors:** Monika Michalak

**Affiliations:** Department of Dermatology, Cosmetology and Aesthetic Surgery, Medical College, Jan Kochanowski University, 35-317 Kielce, Poland; monika.michalak@ujk.edu.pl

**Keywords:** plants, skin, photoprotection, wound healing, anti-aging, anti-tyrosinase, essential oils, colorants, cosmetics, pharmaceutics

## Abstract

Natural ingredients have been used for centuries for skin treatment and care. Interest in the health effects of plants has recently increased due to their safety and applicability in the formulation of pharmaceuticals and cosmetics. Long-known plant materials as well as newly discovered ones are increasingly being used in natural products of plant origin. This review highlights the beneficial effects of plants and plant constituents on the skin, including moisturizing (e.g., *Cannabis sativa*, *Hydrangea serrata*, *Pradosia mutisii* and *Carthamus tinctorius*), anti-aging (e.g., *Aegopodium podagraria*, *Euphorbia characias*, *Premna odorata* and *Warburgia salutaris*), antimicrobial (e.g., *Betula pendula* and *Epilobium angustifolium*), antioxidant (e.g., *Kadsura coccinea*, *Rosmarinus officinalis*, *Rubus idaeus* and *Spatholobus suberectus*), anti-inflammatory (e.g., *Antidesma thwaitesianum*, *Helianthus annuus*, *Oenanthe javanica*, *Penthorum chinense*, *Ranunculus bulumei* and *Zanthoxylum bungeanum*), regenerative (e.g., *Aloe vera*, *Angelica polymorpha*, *Digitaria ciliaris*, *Glycyrrihza glabra* and *Marantodes pumilum*), wound healing (e.g., *Agrimonia eupatoria*, *Astragalus floccosus*, *Bursera morelensis*, *Jatropha neopauciflora* and *Sapindus mukorossi*), photoprotective (e.g., *Astragalus gombiformis*, *Calea fruticose*, *Euphorbia characias* and *Posoqueria latifolia*) and anti-tyrosinase activity (e.g., *Aerva lanata*, *Bruguiera gymnorhiza*, *Dodonaea viscosa*, *Lonicera japonica* and *Schisandra chinensis*), as well as their role as excipients in cosmetics (coloring (e.g., *Beta vulgaris*, *Centaurea cyanus*, *Hibiscus sabdariffa* and *Rubia tinctiorum*), protective and aromatic agents (e.g., *Hyssopus officinalis*, *Melaleuca alternifolia*, *Pelargonium graveolens* and *Verbena officinalis*)).

## 1. Introduction

**Figure 1:**
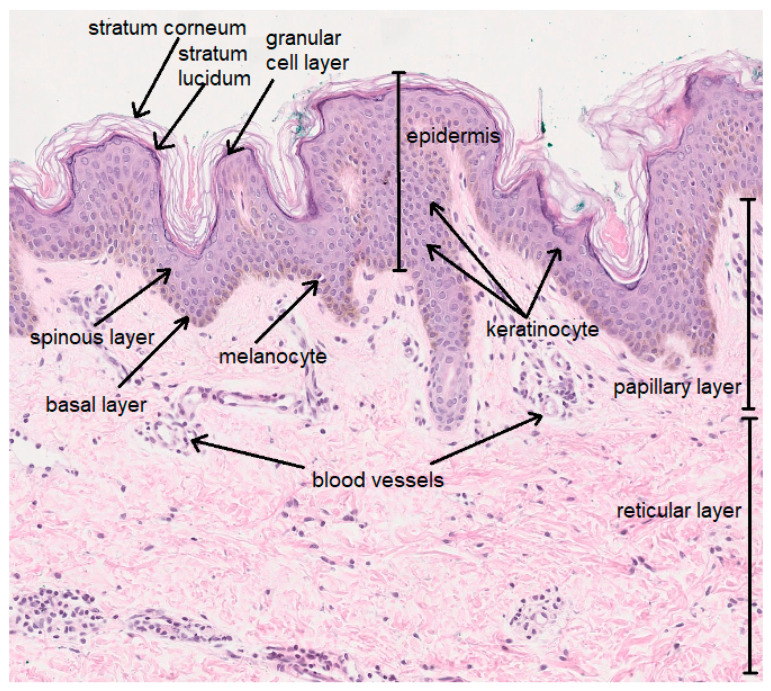
Structure of the skin (own work; photo: Department of Clinical and Experimental Pathology, Medical College, Jan Kochanowski University).

The skin consists of the epidermis and the dermis, below which lies subcutaneous tissue. The five-layer epidermis consists of keratinocytes—cells taking part in keratinization, melanocytes—pigment cells, Langerhans cells, mastocytes and Merkel cells. The dermis is composed of connective tissue and consists of a papillary layer and a reticular layer. It contains fibroblasts, which are responsible for the production of collagen, elastin and glycosaminoglycans (GAG), as well as numerous blood vessels, nerve endings and appendages, including hair follicles and sweat and sebaceous glands (Figure 1). The skin performs multiple complex functions; it takes part in metabolic and homeostatic processes and is responsible for the excretion, selective absorption and storage of substances. In addition, it protects against biological (e.g., microbes), physical (e.g., UV radiation) and chemical factors [1,2].

**Figure 2:**
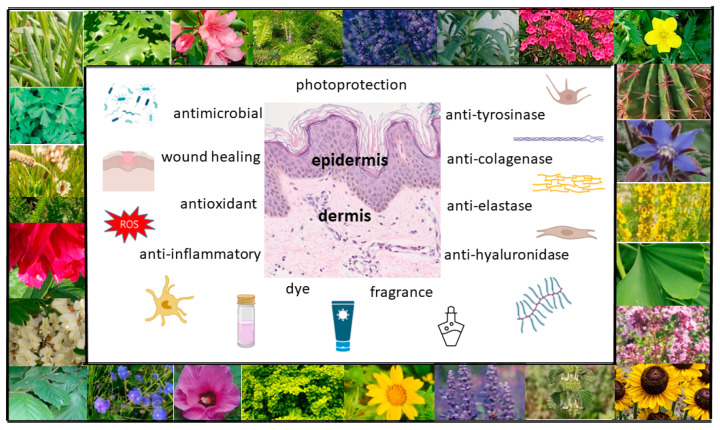
Possible uses of plants in skin care and treatment (own work; photos: M. Michalak).

Botanical ingredients are one of the main sources of materials that are used in the cosmetics and pharmaceutical industries. Recent years have seen increasing interest in dermocosmetics and cosmeceuticals produced from plant materials, and thus, there has been greater interest in plant-based products with skin care properties. Plant materials can be applied topically for skin care purposes, as well as for the treatment of numerous skin diseases [2] (Figure 2). Their advantage is that they are gentle but effective, safe and non-toxic, without side effects. Cosmetics fortified with bioactive compounds are ideally suited to the needs of the skin and are more environmentally friendly than conventional cosmetics. A group of natural ingredients widely used in cosmetics is plant extracts, which are a rich source of biologically active substances significantly affecting human skin. They may exhibit a wide range of properties, both medicinal (in certain skin disorders, including inflammatory disorders such as acne, psoriasis or atopic dermatitis) and for use in skin care (e.g., antioxidant, antibacterial, astringent, moisturizing, regenerating, cleansing, smoothing or lightening) [3,4]. Plant extracts are obtained via extraction from various parts of raw plants, e.g., using an appropriately chosen solvent, such as water, ethyl alcohol, glycerine, glycols or vegetable oil. Plant extracts are obtained from whole plants or parts of plants (fruits, leaves, roots, bark, stems, branches, seeds or flowers). The composition and properties of plant extracts, which can be found in the formulas of natural cosmetics, depend on a variety of factors, including cultivation and harvest conditions, how and to what extent the material is broken up, or drying and extraction methods. Extracts from whole plants as well as individual chemical substances contained in them are used in cosmetics. Active plant substances are divided into primary and secondary metabolites. The former are basic substances that are essential to the plant for life, constituting building materials and energy sources. They include sugars, fats, proteins, amino acids and enzymes. Secondary metabolites include terpenes, steroids, saponins, tannins, alkaloids, volatile oils, resins, vitamins and phenolics [1,4].

The aim of the present paper is to describe plants as bioactive cosmetic and therapeutic substances. This review focuses on recent studies on the potential uses of plants and their constituents as photoprotective, anti-inflammatory, regenerative, wound-healing, anti-aging, depigmenting, aromatic and coloring agents.

## 2. Plants as Photoprotective Agents against Ultraviolet-Radiation-Induced Inflammation and Skin Damage

Ultraviolet (UV) radiation is a physical inflammatory, mutagenic and carcinogenic reagent, as well as a strong enhancer of reactive oxygen species (ROS) production. The biological effects of UV radiation on the skin may be the result of early reactions (erythema or sunburn) or long-term reactions (changes related to skin damage at the molecular and biochemical level). The first response of the skin to UV radiation is the activation of inflammation. UVB irradiation of keratinocytes leads to increased synthesis of pro-inflammatory cytokines in the epidermis, e.g., TNF-α (tumour necrosis factor α) and interleukins IL-1, IL-6, IL-8 and IL-10, which then influence immune cell activity. Another important mediator of inflammation induced by UV radiation is cyclooxygenase-2 (COX-2). COX-2 is an enzyme that is responsible for the synthesis of prostaglandins (PG) from arachidic acid; these play an important role in the regulation of the inflammatory reaction of skin exposed to UVB radiation [5,6,7,8]. Moreover, skin cells exposed to UV radiation respond by activating a cascade of signaling pathways. Disruptions in the activation of these pathways induced by UV radiation lead to disturbances of the homeostasis of the skin, changes in gene expression or the regulation of cytokine secretion, or a loss of control over the cell cycle, which in turn can lead to carcinogenesis [9]. Key signaling pathways activated by UV radiation include transcription factor NFκB (nuclear factor of kappa in B cells) and MAPKs (mitogen-activated protein kinases), including p38 kinases (p38 mitogen-activated protein kinases), JNK (Jun N-terminal kinase) and ERK 1/2 (extracellular signal-regulated kinase 1/2). The p38 kinase is activated by a number of pro-inflammatory cytokines or stress factors. Studies suggest that the p38 kinase is involved in the activation of inflammation induced by UVB radiation through the regulation of COX-2 activity, the production of IL-6, IL-8 and TNFα and the synthesis of nitric oxide (iNOS). Studies have shown that the JNK serine-threonine kinase pathway is more strongly activated by UVA radiation than by UVB radiation in human keratinocytes. The type and dosage of UV radiation have also been shown to determine the activation of ERK1/2 [10,11,12,13]. The exposure of human keratinocytes to UV radiation results in ROS accumulation. Oxidative stress may modulate various signaling cascades in human skin cells and mediate MAPK activity, and it may also be associated with elevated levels of activator protein 1 (AP-1) and NFκB in keratinocytes. Prolonged and intense exposure to UV radiation contributes not only to premature skin aging but also to melanoma and nonmelanoma skin cancers (cutaneous malignant melanoma, basal cell carcinoma or squamous cell carcinoma) [2,14].

Selected plant extracts and single compounds with antioxidant, anti-inflammatory and immunomodulatory effects play an important role in the photoprotection of the skin. Phytochemicals have shown the ability to act as free radical scavengers, radical chain reaction inhibitors, metal chelators, oxidative enzyme inhibitors and antioxidant enzyme cofactors. Some studies have reported that plant extracts promote endogenous antioxidant enzymes such as catalase (CAT), superoxide dismutase (SOD) and glutathione peroxidase (GSH-PX), which protect the skin against increasing ROS levels under oxidative stress. Moreover, plant materials can modulate the expression and activation of a wide variety of cytokines, such as TNF-α IL-1β, IL-6 and IL-8. Botanicals have also shown the ability to regulate the expression of various pro-inflammatory genes and inhibit the activity of pro-inflammatory enzymes such as inducible nitric oxide synthase (iNOS), COX-2 and lipoxygenase (LOX) [15,16,17].

Plant extracts and natural compounds from plants have been reported in the earlier literature to possess photoprotective properties. These include phytochemicals such as ferulic, caffeic, cinnamic, rosmarinic acid, quercetin, apigenin, rutin, luteolin, chrysin, hesperidin, dihydromyricetin, chrysanthemin, curcumin, genistein, resveratrol, carnosic, ursolic, ellagic, asiatic acid, zerumbone, astaxanthin, β-carotene, lycopene, zeaxantin, lutein and L-ergothioneine, as well as extracts from plants such as *Opuntia humifusa* [18], *Camellia sinensis* [19], *Punica granatum* [20], *Hibiscus furcatus*, *Atalantia ceylanica*, *Mollugo cerviana*, *Leucas zeylanica*, *Ophiorrhiza mungos*, *Olax zeylanica* [21] *Silybum marianum* [22], *Polypodium leucotomos* [23], *Vaccinium myrtillus* [24], *Lonicera caerulea* [25], *Thymus vulgaris* [26], *Opuntia ficus-indica* [27], *Morinda citrifolia* [28], *Galinsoga parviflora*, *Galinsoga quadriradiata* [29], *Coffea arabica* [30], *Amaranthus cruentus*, *Moringa oleifera*, *Malaxis acuminata*, *Schinus terebinthifolius* [31], *Schinopsis brasiliensis* [32], *Crataegus pentagyna* [33], *Sambucus nigra*, *Helichrysum arenarium*, *Crataegus monogyna* [34], *Capnophyllum peregrinum* [35], *Dalbergia* monetaria [36], *Baccharis antioquensis* [37], *Juglans regia* [38], *Dimorphandra gardneriana* and *Lippia microphylla* [39].

Some plants that are effective UV filters may be potential sunscreen ingredients [40]. These include plant extracts such as *Astragalus gombiformis* with an SPF value of 38 [41], *Sloanea calva* with an SPF value of 35.4 [42], *Hylocereus polyrhizus* with an SPF value of 35.02 [43] or *Rosa centifolia* with SPF values of 32 [44]. Moreover, plant extracts, through their synergistic effects with some physical or chemical UV filters (e.g., benzophenone-3 (BP-3), octyl methoxycinnamate (OMC) or titanium dioxide (TiO_2_)), may also play a role as cosmetic components that enhance the SPF of sunscreen formulations [40]. This effect has been shown for extracts from *Sanionia uncinata* [45,46], *Vitis vinifera* [47], *Nephelium lappaceum* [48], *Psidium guajava* [49], *Campomanesia adamantium* and Campomanesia xanthocarpa [50], as well as moss extracts from *Leucobryum* spp. and *Holomitriopsis laevifolia* [51].

Table 1 presents the results of research from the last five years on the protective effects of plant-derived products on UVB-mediated damage, with potential applications in photoprotective products.

## 3. Plants as Regenerative and Wound-Healing Agents

**Figure 3:**
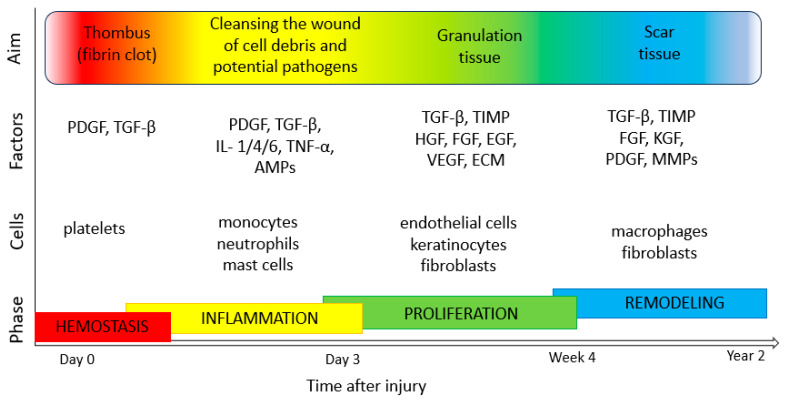
Phases of wound healing (own work based on [82,89,90,91]). TGF-β, transforming growth factor β; PDGF, platelet-derived growth factor; IL-1, 4, 6, interleukin-1, -4, -6); TNF-α, tumor necrosis factor α; AMPs, antimicrobial peptides; TIMP, tissue inhibitors of metalloproteinase; HGF, hepatocyte growth factor; FGF, fibroblast growth factor; EGF, epidermal growth factor; VEGF, vascular endothelial growth factor; KGF, keratinocyte growth factor; ECM, extracellular matrix; MMPs, matrix metalloproteinases.

The process of the regeneration and healing of the skin involves interactions between many types of cells, including endothelial cells, inflammatory cells, keratinocytes and fibroblasts. It consists of stages such as coagulation (haemostasis, fibrin clot formation and activation of the clotting cascade by platelets), inflammation (neutrophil and monocyte migration, phagocytosis of bacteria and the release of proteolytic enzymes to debride the wound), proliferation (angiogenesis by endothelial cells, granulation tissue formation by fibroblasts and reepithelialization by keratinocytes) and tissue maturation (collagen/ECM remodeling by fibroblasts) [82,83,84] (Figure 3). An important step in tissue formation, repair and the maintenance of good skin conditions is proper cell proliferation and migration processes. These depend on many factors, such as biochemical communication, adhesion strength and mechanical flexibility, as well as organization of the cellular cytoskeleton [85,86,87]. Numerous regulators take part in keratinocyte migration and proliferation, including epidermal growth factor (EGF), insulin-like growth factor 1 (IGF-1), fibroblast growth factor (FGF), granulocyte-macrophage colony-stimulating factor (GM-CSF), angiopoietin-related growth factor (AGF), vascular endothelial growth factor (VEGF), transforming growth factor β (TGF-β), connective tissue growth factor (CTGF), platelet-derived growth factor (PDGF) and platelet derived-endothelial cell growth factor (PD-ECGF). In addition, cytokines (e.g., IL-1, IL-6 and TNF-α), neuropeptides (G protein-coupled receptor (GCRP), vasoactive intestinal peptide (VIP) and substance P (SP)), MMPs and extracellular macromolecules also play various roles in the regulation of skin cell motility and proliferation [88,89].

A wound is an injury involving a breach of the integrity of the skin. A chronic wound may lead to complications, such as bacterial infections. Bacterial infections also delay the wound-healing process, prolonging inflammation. The surface of human skin is colonized by commensal bacteria with low virulence, such as coagulase-negative staphylococci and non-pathogenic corynebacteria and cutibacteria, but also by opportunistic pathogenic microbes (such as *Candida* spp., *Malassezia* spp. or *Staphylococcus aureus*) and bacteria with high pathogenic potential (e.g., *Streptococcus pyogenes*). The skin of hospitalized patients who have undergone antibiotic treatment may be colonized by Gram-negative non-fermenting bacteria (*Pseudomonas aeruginosa* or *Acinetobacter baumannii*) or yeasts, including the opportunistic pathogen *Candida auris.* The choice of treatment for skin and wound infections depends on various factors (e.g., the severity of the disease or host factors), but plants and drugs of natural origin can undoubtedly have broad applications alongside topical synthetic antibiotics and antiseptic agents [92,93,94].

Botanicals have been used topically for decades for skin regeneration and the treatment of dermatological problems, such as chronic diabetic wounds, ulcers, bedsores, burns and non-healing wounds. Numerous plants and drugs of natural origin support the normal repair systems of the skin and therefore show great therapeutic potential in skin regeneration and wound treatment by various mechanisms. These include effects on keratinocyte migration and proliferation rates, modulation of the release of various growth factors, cytokines, chemokines or neuropeptides by skin cells, increasing the formation of capillary vessels and increasing fibroblast activity. Another important group of raw materials comprises plants with astringent and antimicrobial properties, which contribute to wound contraction and increase the rate of epithelialization [83,84,95]. The scientific literature points to the important effects of plants (e.g., *Achiella millefolium* [96], *Aloe vera* [97], *Althaea officinalis* [98], *Calendula officinalis* [99], *Curcuma longa* [100], *Eucalyptus globulus* [101], *Simmondsia chinensis* [102], *Pinus sylvestris* [103] and *Camellia sinensis* [104]) and phytochemicals (e.g., triterpenes, alkaloids and flavonoids) on tissues and their potential to amplify skin regeneration and accelerate the process of wound repair and healing [84,95].

Table 2 cites some original research carried out in the last five years on selected plants and their constituents, as well as formulations based on raw materials of plant origin exhibiting wound-healing activity and potential applications in regeneration and skin treatment.

## 4. Plants as Anti-Aging Agents

Preventing and combating signs of skin aging (dry skin, loss of firmness and elasticity or wrinkles) is an age-old challenge. The skin is the organ on which these processes are most noticeable, hence the great interest in age-related changes at the level of the epidermis, dermis and subcutaneous tissue. At the level of the epidermis, changes observed with age include (1) thinning of all layers of the epidermis and flattening of the dermo-epidermal junction; (2) disturbances of the production of natural moisturizing factor (NMF), leading to dryness and increased peeling of the epidermis; (3) a reduction in the level of epidermal lipids (mainly ceramides); and (4) oxidation of lipids of intercellular cement, leading to increased transepidermal water loss (TEWL) [144,145]. Disturbed production of lipids binding the corneocytes of the stratum corneum not only causes skin dryness but also disrupts the process of the exfoliation of keratinized epidermal cells. This is linked to the malfunction of enzymes, enabling exfoliation when the water content in the epidermis is low. For example, a deficiency of linoleic acid, a component of ceramide 1, with an important role in the cohesiveness of cement, is associated with dry skin symptoms [146,147]. Major age-related changes in the dermis include (1) a reduced number and activity of fibroblasts, which are cells that are responsible for the synthesis of collagen fibers, elastin fibers and hyaluronic acid; (2) degradation of collagen fibers, progressive collagen cross-linking and a reduction in skin resilience and resistance to stretching; (3) changes in the structure of elastin fibers, which clump together in an amorphous mass (elastosis), loss of elasticity and wrinkle formation; and (4) a reduction in hyaluronic acid, with insufficiently moisturized and resilient skin [144,145].

Over the centuries, the search for new substances to slow down the aging process and restore the skin’s young appearance has not diminished. Bioactive substances with anti-aging properties include moisturizers, which influence the hydrolipid barrier and minimize destructive lesions occurring in the stratum corneum. The skin may be hydrated through the external supply of water from moisturizing agents or via the application of agents forming an occlusive lipid film to slow down water loss from the skin. An important group of anti-aging agents comprises bioactive substances, which take part in the synthesis and metabolism of skin components (e.g., proteins and essential unsaturated fatty acids) and also exhibit collagenase, elastase and hyaluronidase inhibitory activity [1,144]. Collagenase is an enzyme belonging to the family of matrix metalloproteinases (MMP), which can degrade collagen, the fibrous component of the extracellular matrix (ECM) and the major structural protein in human skin. Elastase is a proteolytic enzyme involved in the degradation of elastin, a protein responsible for skin elasticity. Hyaluronidase is an enzyme (an endoglycosidase) responsible for the hydrolysis of hyaluronic acid, a skin glycosaminoglycan, which is a major component of ECM [148,149].

Botanicals that support the health, texture and integrity of the skin are widely used in cosmetic formulations for dry and mature skin. Plant extracts and natural products are recommended because they increase skin hydration, reduce TEWL, display skin-barrier-reinforcing properties, inhibit the degradation of skin components and help to maintain the integrity of the skin’s structure. These are promising approaches to preventing skin aging using products derived from plants. Plants can be a very interesting source of ingredients with potential anti-aging properties, as confirmed by the results of in vitro studies. However, further research is needed to confirm the efficacy of plant-derived materials in vivo, as the most important factor determining the effectiveness of active ingredients of natural origin is their bioavailability. In some studies, plants have been shown to exert notable in vivo anti-aging properties. According to the literature, skin parameters associated with skin aging, such as skin hydration (measured with a corneometer and tewameter), skin elasticity (measured with a cutometer and elastometer) or facial wrinkles (measured with a skin visiometer and camera for skin analysis) have been evaluated following the application of cosmetic formulations based on various plant extracts, alone or in combination [119,150,151,152]. Table 3 cites research from the last five years on selected plant species and their extracts with potential uses as agents preventing and slowing down skin aging.

## 5. Plants as Anti-Tyrosinase Agents

**Figure 4:**
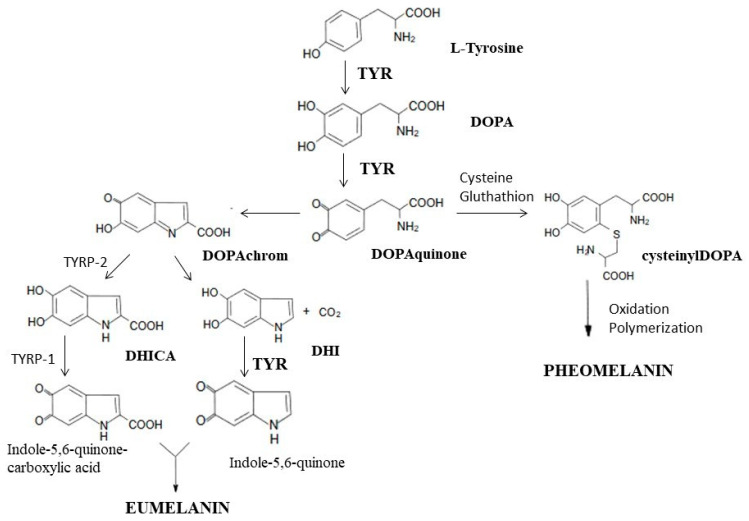
Participation of tyrosinase in the synthesis of melanins: eumelanin and pheomelanin. TYR, tyrosinase; DOPA, dihydroxyphenylalanine; TYRP2, tyrosinase-related protein 2; TYRP1, tyrosinase-related protein 1; DHICA, 5,6-dihydroxyindole-2-carboxylic acid; DHI, 5,6-dihydroksyindol (own work based on [170,172,174]).

Tyrosinase is an enzyme that is widely distributed in the cells of animals, plants and microorganisms. It is a key enzyme in the biosynthesis of melanin, responsible for the catalysis of the first two synthesis reactions, i.e., the hydroxylation of tyrosine to DOPA and the oxidation of DOPA to dopaquinone. At the stage of dopaquinone formation, the eumelanin and pheomelanin pathways are separated. When thiol compounds (cysteine and glutathione) are present, they attach to dopaquinone, and the biosynthesis pathway is redirected toward pheomelanin. When the L-tyrosine concentration is low and that of cysteine is high, cysteine attaches to dopaquinone, and cysteinyldopa isomers are formed [170,171]. In the absence of thiol compounds, highly reactive dopaquinone easily undergoes intracellular cyclization, oxidation and transformation to dopachrome [170,171,172,173]. In the presence of TYRP2 (tyrosinase-related protein 2, also called dopachrome tautomerase—DCT) or metal cations (Cu^2+^, Zn^2+^, Fe^2+^, Co^2+^ or Ni^2+^), dopachrome may be converted to 5,6-dihydroxyindole-2-carboxylic acid (DHICA) [170,173]. In the absence of DCT, dopachrome is converted to 5,6-dihydroxyindole (DHI) by nonenzymatic decarboxylation [170]. TYRP1 (tyrosinase-related protein 1) causes the oxidation of DHICA to indole-5,6-quinone-2-carboxylic acid, and TYR causes the oxidation of DHI to indole-5,6-quinone. The polymerization of the resulting monomers (indole and quinone) leads to the formation of eumelanin [171,173] (Figure 4).

As a metalloenzyme, tyrosinase has two copper atoms in its active site, determining its catalytic function. Substances belonging to the group of tyrosinase inhibitors inhibit melanin synthesis by interacting with copper ions in the active site of tyrosinase, thereby reducing the activity of the enzyme [175,176].

In recent years, anti-tyrosinase agents have attracted the attention of researchers searching for substances that can whiten the skin and also treat skin pigmentation disorders. Ongoing research indicates that many plant extracts and plant-derived chemicals are strong tyrosinase inhibitors and prevent the overproduction of melanin in the epidermal layers. At the same time, importantly, they inhibit melanogenesis without exerting cytotoxic or mutagenic effects on melanocytes [175,177,178,179]. Constituents of plant extracts with depigmenting properties resulting from the inhibition of tyrosinase activity include arbutin (found in, e.g., *Pyrus pyrifolia* peel (3.35 mg/g) [180], *Origanum majorana* herbs (51.3 mg/g) [181], *Arctostaphylos uva*-*ursi* leaves (6.4%) [182], *Vaccinium vitis idaeae* leaves (46.78 mg/g) [183] or *Bergenia crassifolia* leaves (22.59%) [184]), coumaric acid (present in, e.g., *Artocapus altilis* fruits (11.85 mg/100 g) [185,186]), ellagic acid (occurs in, e.g., *Juglans regia* leaves (16.25%), *Castanea sativa* stem bark (2.75%) or *Eucalyptus camaldulensis* leaves (0.28%) [187]), aloesin (isolated from the *Aloe vera* leaves (64 mg/L) [188]), baicalein (present in *Scutellaria baicalensis* roots (16.61 mg/g) [189,190]) and glabridin (found in *Glycyrrhiza glabra* roots (22.87 mg/g) [191]).

Table 4 presents research from the last five years on various plant species tested for anti-tyrosinase activity with potential uses in products for depigmenting or lightening the skin.

## 6. Plants as Aromatic Agents

Over the centuries, the aromatic applications of plant extracts have gained importance. Plant essential oils, considered to be those with an oil content above 0.01% of the fresh weight of the plant, are of particular importance. Some plant materials may contain even 20% essential oils (EOs) [256,257,258]. EOs are mainly obtained from plants of the Apiaceae, Asteraceae, Lamiaceae, Lauraceae, Myrtaceae, Rutaceae, Verbenaceae and Geraniaceae families [257,259] (Table 5). EOs can be found in all parts of the plant, i.e., the flowers (rose, lavender, jasmine or ylang-ylang), leaves (eucalyptus, peppermint, geranium, rosemary or tea tree), herbs (basil, hyssop and lemon balm), roots (ginger and vetiver), wood (cedarwood, camphor and sandalwood), bark (cinnamon and myrtle), seeds (anise, cumin, cardamom and fennel) and fruits (pepper, nutmeg and juniper). They are obtained from raw plant materials via distillation (water, steam or dry distillation), extraction (microwave, ultrasound, solvent extraction, maceration or enfleurage) or mechanical or cold pressing. EOs are mixtures of volatile substances, mostly colorless or light yellow, with an intense odor and an oily consistency, and they are soluble in liquid fats, alcohol, ether or chloroform. The biological activity and fragrance of EOs are determined according to their chemical composition. Their composition depends on numerous factors, including the origin of the plant materials or the conditions of plant growth. EOs are not chemically homogeneous. They may contain up to several hundred chemical compounds, including terpene hydrocarbons and their oxygen derivatives, alcohols, aldehydes, ketones, organic acids, esters and ethers [256,257,259,260]. Some compounds of EOs have a characteristic aroma, e.g., bisabolol, with a sweet floral odor; geraniol, with a fresh, sweet and rose-like odor; linalyl acetate, with a floral, sweet citrus odor; citronellol, with a strong floral, rose-like and sweet odor; limonene, with a strong orange odor; linalool with a floral, grassy, pleasant and citrus odor; myrcene, with a pleasant floral odor; terpineol, with a sweet, lilac odor; α-pinene, with a fresh, camphor, sweet and pine odor; or β-phellandrene, with a mint, turpentine odor [260].

Cosmetic aromatherapy utilizes EOs for skin, body, face and hair products. EOs are added to skincare and bath cosmetics or massage preparations as substances providing fragrance and as active ingredients. Smell is an important criterion in purchasing cosmetic products. A wide range of essential oils is available, and their marketing potential is enormous. Fragrance composition is an important element of the formulation of new cosmetic preparations. Fragrances also play an important role in masking unpleasant aromas from fatty acids, oils and surfactants used in cosmetic formulations [256,258,260].

EOs and their constituents, in addition to their aromatic effects, are also used in modern cosmetics and dermocosmetics as absorption promoters and preservatives [258]. The absorption of active substances by the skin can also be increased by EOs, such as eucalyptus, peppermint or terpentine oil, as well as by components of essential oils, such as menthol, limonene, carvacrol, linalool, α-pinene or terpineol [258,259]. Due to their antimicrobial action, EOs can act as natural preservatives to prolong the durability of cosmetics, e.g., essential oils from lavender (*Lavandula angustifolia*) [261], thyme (*Thymus vulgaris*) [263], peppermint (*Mentha piperita*) [264], cajuput (*Melaleuca cajuputi*), cinnamon (*Cinnamomum zeylanicum*) [271], clove (*Syzygium aromaticum*) [275], eucalyptus (*Eucalyptus globulus*) [273], sage (*Salvia officinalis*) [277] and tea tree (*Melaleuca alternifolia*) [274]. EO constituents performing this function include phenols, aldehydes, alcohols, ketones and esters [258,259].

The use of EOs may have side effects, such as allergic reactions, irritation or temporary sensitivity to UV radiation. An allergic reaction or skin irritation may occur following the use of cinnamon, clove or lemon grass oil, and oils with a photosensitizing effect include citrus oils (e.g., bergamot, lime, bitter orange, lemon or grapefruit), as well as EOs present in angelica root (*Angelica archangelica*), rue (*Ruta graveolens*), parsley leaf (*Petroselinum crispum*) and marigold (*Tagetes minuta*). Constituents of EOs that may trigger allergic reactions include benzyl alcohol, cinnamyl alcohol, eugenol, hydroxycitronellal, isoeugenol, benzyl salicylate, cinnamaldehyde, coumarin, geraniol, anisyl alcohol, benzyl cinnamate, farnesol, linalol, benzyl benzoate, citronellol or limonene [258,259,260]. EO safety in the cosmetic industry is monitored in a variety of ways, e.g., by the International Fragrance Association (IFRA) and the International Organization for Standardization (ISO) [260].

## 7. Plants as Colorants and Dye Agents

The history of the human use of pigments dates back to prehistoric times. Dye plants that are known to have been used in various periods include dyer’s madder (*Rubia tinctorum*), true indigo (*Indigofera tinctoria*), dyer’s woad (*Isatis tinctoria*), dyer’s weed (*Reseda luteola*) and logwood (*Haematoxylum campechianum*) [278]. Dyes that are currently used in cosmetics were once used in various branches of industry. It is believed that dyes were originally used for ornamental purposes. In ancient Egypt, mainly the skin and hair were dyed, e.g., using henna (a pigment obtained from the shrub *Lawsonia inermis*). In modern cosmetology, plant pigments are added to cosmetic products to give them an aesthetic appearance. Like aroma, color plays an important role in marketing cosmetics and pharmaceutical products [278,279,280]. In addition, colorants and dyes are used as beauty enhancers, masking imperfections or correcting minor skin defects. Apart from color cosmetics (e.g., fluids, lip pencils, lipstick, rouge or eyeshadow), plant pigments are also a component of skin care cosmetics with protective and antioxidant properties, with the ability to strengthen blood vessels and improve the condition of skin [281,282].

Plant dyes, which are varied in terms of chemical structure, are a group of compounds that are present in plant parts such as flowers, fruits and leaves. Plant pigments include quinones, polyphenols, chlorophylls, carotenoids and betalains [279,281,282,283,284] (Table 6).

Quinones are compounds whose color ranges from yellow to orange to red to brown. Quinones, which include benzoquinones, naphthoquinones and anthraquinones, are a large group of pigments. Anthraquinones are anthracene derivatives that are widespread in the plant world. They can be found among plants of the Polygonaceae, Rubiaceae, Rhamnaceae, Scrophulariaceae, Liliaceae, Hypericaceae and Fabaceae families. In traditional dyeing, hypericin, a red dye obtained from St John’s wort (*Hypericum perforatum*), was used as well. Natural fibers were also dyed using rhamnotoxin—a red pigment obtained from the bark of alder buckthorn—as well as with alkannin, from the rhizomes and roots of dyer’s alkanet (*Alkanna tinctoria*). This dye has been used since ancient times in color cosmetics, such as lipsticks. Another source of alkannin, which is a naphthoquinone derivative, is the root of common bugloss (*Anchusa officinalis*) [279,281,282].

A wealth of flavonoids can be found in plants of the Apiaceae, Asteraceae, Betulaceae, Polygonaceae, Brassicaceae, Ericaceae, Fabaceae, Hypericaceae, Primulaceae, Lamiaceae, Rosaceae, Rubiaceae, Rutaceae and Scrophulariaceae families. Apart from their role in skin care, flavonoids are used in cosmetics as natural plant dyes, including flavonols (intense yellow), flavones (light yellow and cream-colored), chalcones (light yellow) and aurones (intense yellow) [279,281,282].

Anthocyanins are widespread plant dyes, the most common of which include red pelargonidin (geranium and dahlia), blue-to-red peonidin (elderberry and peony) and cyanidin (cornflower, chokeberry, cranberry and cherry), purple malvidin (mallow and grapes), petunidin (petunia) and delphinidin (grape, elderberry and cranberry). Tannins are broadly distributed in the plant kingdom and are generally classified into two types: hydrolysable tannins (e.g., gallotannins and ellagitannins) and condensed tannins (catechins and leucoanthocyanidins). Plants supplying brown, gray or sometimes rust-colored tannin dyes include the species *Uncaria gambir*, *Galla chinensis* (Chinese gallnut), *Acacia catechu*, *Schinopsis balansae*, *Pteropcarpus marspinum*, *Eucalyptus rostrata*, *Quercus infectoria*, *Quercus robur*, *Quercus sessilis*, *Potentilla erecta*, *Alchemilla vulgaris*, *Sanguisorba officinalis* and *Polygonum bistorta* [279,281,282,285].

Chlorophylls are a pigment that is present in all green plants (in the stems, leaves, flowers, fruits or seeds), e.g., *Urtica dioica*, *Medicago sativa*, spinach, lettuce and broccoli. Among the known plant chlorophylls, two have significance as dyes: chlorophyll *a* (blue-green) and chlorophyll *b* (yellow-green). Chemically, chlorophyll is an ester (magnesium porphyrin composed of four pyrrole rings) with two alcohols (phytol and methanol) [280,282].

Carotenoids are polyene dyes, i.e., they have a conjugated double-bond system. Plant sources of carotenoids include *Crocus sativus*, from which the stigma, containing the yellow carotenoid pigment crocin, is used; *Bixa orellana*, whose fruits supply the yellow-orange carotenoid pigment bixin (annato, orlean); and *Calendula officinalis*, whose flowers contain α- and β-carotene, lutein, lycopene and violaxanthin [281,282,286].

Betalains are found in plants of the order Caryophyllales. Sources of betalain pigments include beet root (*Beta vulgaris*), the fruits of the prickly pear *(Opuntia ficus-indica)* or cacti of the *Hylocereus* genus and the flowers of numerous species of the Amaranthaceae family [281,282].

**Table 6:** Classification of natural colorants according to chemical functional groups (structure) [279,282,287].

	Chemical Class	Example of Class	Source	Color Produced
Quinones	benzoquinone	1,4-benzoquinone	*Pyrus lindleyi*	brown
	anthraquinones	alizarin	*Rubia tinctiorum*	red
	napthoqinones	lawsone (2-hydroxy-1,4-naphthoquinone)	*Lawsonia inermis*	brown, purple grey and shades of orange
		juglone (5-hydroxy-1,4-naphthoquinon)	*Juglans regia*	
Polyphenols	flavones	luteolin	*Reseda luteola*	yellow and brown
		apigenin	*Chamomilla recutita*	
		chrysin	*Passiflora incarnata*	
	anthocyanins	protocyanins	*Centaurea cyanus*	red, violet or blue (depending on pH)
		malvidin, peonidin, delphinidin	*Althaea rosea*	
		3-delphinidin sambubioside (hibiscin), 3-cyanidin sambubioside, 3-delphinidin glucoside	*Hibiscus sabdariffa*	
		3-cyanidin glucoside (chrysanthemum), 3-cyanidin sambubioside	*Sambucus nigra* *fructus*	
Betalains	betacyanins	betanin	*Beta vulgaris* *Amaranthus cruentus* *Opuntia ficus-indica*	red and purple
	betaxanthins	vulgaxanthin I and II, indicaxanthin	*Hylocereus* polyrhizus, *Opuntia ficus-indica*, *Beta vulgaris*	yellow and orange
Carotenoids	carotenes	α-, β-, γ-carotene, lycopene	*Daucus carota*, *Solanum lycopersicum*, *Sorbus aucuparia*	orange, red and yellow
	xanthophylls	lutein, zeaxanthin, violaxanthin	*Spinacia oleracea*, *Zea mays*, *Tagetes erecta*	

Natural colorants and dyes of plant origin have the important advantages of being nontoxic, safe, without side effects, non-carcinogenic, environmentally friendly (biodegradable and compatible with the environment) and economical. For these reasons, they are becoming an object of consumer interest with broad applications in the cosmetic industry. Plant dyes can be an alternative to synthetic dyes, which involve the use of petrochemical-based materials, and due to their allergic, toxic, mutagenic, genotoxic and carcinogenic effects, they are responsible for various health and skin problems [280,283,287].

## 8. Future Perspectives and Challenges

In the European Union, before cosmetic products can be sold to customers, they must be evaluated for safety in accordance with Regulation (EC) No. 1223/2009 of the European Parliament and of the Council, and in the United States, the safety of cosmetics is regulated by the Food and Drug Administration (FDA), mainly through the Federal Food, Drug, and Cosmetics Act (FD&C Act) and the Fair Packaging and Labeling Act (FPLA). The global cosmetics industry (encompassing products for the face, eyes, hair, nails, mouth and body, which may be used externally for cleansing, beautifying or altering one’s appearance) is continually growing, together with consumer awareness regarding health care, including hygiene and skin care [288]. Among the entire range of cosmetics, plant-based products have seen tremendous growth of about 15–20 per cent over the past five years. This review presents a wide assortment of plants with various applications in cosmetic preparations that have been reported in the last five years. It is also important to consider certain aspects of the use of plants and bioactive compounds of plant origin in cosmetics and the associated challenges.

First, attention should be paid to the ability of active ingredients of natural origin to penetrate the first skin barrier, as the bioavailability of bioactive compounds is an important factor determining their effectiveness. One promising solution for the future is the development of delivery systems for bioactive ingredients that facilitate penetration, through improved encapsulation and targeted delivery. A related issue is the fact that the effects of these agents have not been conclusively demonstrated in all cases. For example, although some natural agents appear to have promising sun-protection effects, when they are added to sunscreens, this effect has been shown to be poor and to ensure only a modest or low increase in SPF (e.g., lycopene [289] and *Cucumis sativus* extract [290]). Therefore, in vitro research into the biological activity of plants must also be supported by in vivo studies. Even when preliminary studies show promising effects, confirmation in clinical trials is needed.

Second, it is important to consider the mechanism of action and the safety of plant-derived bioactive ingredients. A good example is bergamot oil. The use of methoxypsoralens from the *Citrus bergamia* essential oil following sun exposure has been shown to increase photosensitivity, causing further damage rather than providing photoprotection, despite its stimulating effect on tyrosinase activity [291]. Other adverse effects, such as acute toxicity, skin and eye irritation or skin sensitization, may occur following the topical application of materials of plant origin. This is why it is essential to conduct research not only on the effectiveness of these substances but on their safety as well, prior to including them in a cosmetic formulation.

In addition, discussions about ingredients of plant origin and their biological activity should take into account their chemical structure. One example is the role of flavonoids and their effect on melanogenesis in relation to the chemical structure of this complex group of compounds. For example, hesperetin [292] and genistein [293] have been shown to stimulate melanogenesis, whereas compounds such as epicatechin (EGCG) [294] or baicalein [189] act as inhibitors of melanin formation. It is interesting to compare the two structurally similar compounds apigenin and luteolin. One additional hydroxyl group in luteolin results in different cellular functions: apigenin stimulates melanin synthesis [295], whereas luteolin inhibits it [296]. This suggests that the characteristic chemical structure of individual bioactive compounds leads to differences in how they regulate melanogenesis. Conflicting reports in the scientific literature regarding quercetin may also be puzzling, as some data suggest that it stimulates melanogenesis [297], whereas other data indicate an inhibitory effect against melanogenesis [298]. This demonstrates that there is still a need for in-depth research leading to a better understanding of these plant-derived molecules.

Another important consideration is how the plant material to be used as a cosmetic component is obtained (e.g., the extraction/separation technique, temperature or type of solvent used). Some of the active compounds present in plants (e.g., polyphenols, essential oils or vitamins) have low stability, and their sensitivity to light and heat limits their use in cosmetics. Research in this area is aimed at the development of more stable derivatives or the encapsulation of active substances in liposomes, which protects them from degradation.

The implementation of new solutions for obtaining and preparing plant-derived materials and including them in a cosmetic product is associated with the issue of intellectual property. The mechanisms of the legal protection of innovations, such as patents, are also worthy of attention. Naturally, not all research results can be patented. In the context of plant-based cosmetic materials, no plant or substance extracted from it can be protected by the patent system; however, a complex or mixture of plant extracts or isolated molecules, if it meets the criteria of novelty, inventive activity and industrial application, is patentable [299,300]. Patents involving pharmaceutical and cosmetic applications may refer to the ingredients, formulation, product type, use of pharmaceutical carrier systems or cosmetic production/manufacturing methods [300]. In patents filed in the National Institute of Industrial Property (INPI), types of applications of plant extracts in cosmetics include multifunctional product innovation (e.g., the use of a plant extracts for the treatment of gynoid lipodystrophy and acne), extraction processes used to isolate active ingredients with potential applications in cosmetics and the use of extracts with anti-aging, skin/hair pigmentation and conditioning or photoprotection properties [299,301]. An analysis of patents related to cosmetics containing plant ingredients reveals a high proportion of innovations involving the use of species from the Fabaceae, Asteraceae, Rosaceae, Lamiaceae, Poaceae, Rutaceae, Lilliacae and Apiaceae families [301]. Examples of plants described in patents for cosmetic applications include *Pothomorphe umbellata* root extract for anti-aging activity and the treatment of cell damage caused by exposure to UV rays; *Glycyrrhiza glabra* and *Shophora flavecens* roots for the treatment of skin hyperpigmentation; the *Artemisia* plant species for whitening the skin and delaying aging; or the *Pueraria* plant species for rejuvenation, lightening the skin and treating skin inflammation [299,301].

## 9. Conclusions

Plants and their constituents can be used to maintain the physiological balance of human skin. Ongoing research provides valuable information on the chemical composition and pharmacological properties of botanicals. Moreover, studies have confirmed their effectiveness and have demonstrated new potential applications of plant materials in products for topical use as skin care and therapeutic agents with multifaceted effects. Natural products of plant origin can be used as a safe and efficacious alternative to synthetic products. This is reflected in growing consumer interest in natural cosmetics and the market trend expressed by the development and increasing number of products based on plant-derived ingredients.

## Figures and Tables

**Table 1:** Selected plant extracts from various species and their photoprotective properties.

Species (Family)	Plant Material	Method	Effect	Ref
*Adenocaulon himalaicum* (Asteraceae)	leaf, EE	in vitro, HaCaT exposed to UVB	↑ filaggrin, involucrin, loricrin expression ↓ MMP-1; MAPK, AP-1 activation	[52]
*Alpinia officinarum* (Zingiberaceae)	rhizome, WE	in vivo, UVB-irradiated hairless mouse; in vitro, NIH-3T3 exposed to UVB	↓ of MMP-1 expression and recovered the reduction in collagen content in mouse skin; ↓ IL-6, IL-8, MCP-3 expression and ↓ phospho-Akt and phospho-ERK in NIH-3T3	[53]
*Antidesma thwaitesianum* (Euphorbiceae)	fruit extract	in vitro, UVB-irradiated HaCaT	protects cells from UVB-induced cytotoxicity; anti-inflammatory effect through ↓ NO and ROS generation; ↓ phospho-p38 and phospho-JNK	[54]
*Astragalus gombiformis* (Fabaceae)	aerial part, BE	in vitro, SPF via UV spectroscopy	SPF 37.78	[41]
*Calea fruticosa* (Asteraceae)	aerial part, EE	in vitro, SPF via UV spectroscopy	SPF 9.66	[55]
*Camellia sinensis* (Theaceae)	leaf extract	in vitro, NHEK exposed to UVB	efficacy in recovering TIMP-3 expression downregulated by UVB treatment	[56]
*Chrysophyllum lucentifolium* (Sapotaceae)	ME	in vitro, UVB and H_2_O_2_-treated HaCaT and HDF	↓ expression of COX-2, MMP-1, and -9, HYAL-1, and -4 by downregulating the NF-κB and MAPK (ERK, JNK and p38) pathways; ↑ Col1a1 expression	[57]
*Cistus incanus* *Cistus ladanifer* (Cistaceae)	aerial part extract	in vitro, SPF via UV spectroscopy	SPF 3.33—4.37	[58]
*Ceratonia siliqua* (Fabaceae)	pod and seed extract, WME	in vitro, SPF via UV spectroscopy	SPF 1.07–18.19	[59]
*Corylus avellana* (Betulaceae)	hazelnut skin extract	in vitro, SPF via UV spectroscopy	extract ↑ SPF value of benzophenone 4.66–4.94	[60]
*Cyclopia* spp. (Fabaceae)	leaf and branch, WAE	in vitro, SPF via UV spectroscopy	SPF 27.8	[61]
*Diospyros kaki* (Ebenaceae)	fruit (pulp, skin and seed) extract	in vitro, HaCaT exposed to UVA and UVB	↓ intracellular ROS production in cells; exerts a photoprotective and regenerative effect on UV-irradiated cells	[62]
*Elaeagnus* *angustifolia* (Elaeagnaceae)	leaf extract	in vitro, SPF via UV spectroscopy	SPF values of sunscreen formulation (with 2%, 4%, 6%, 8% extracts): 6.37–21.05	[63]
*Euphorbia characias* (Euphorbiaceae)	leaf, EE	in vitro, SPF via UV spectroscopy	SPF 9.10	[64]
*Helianthus annuus* (Asteraceae)	flower, EE	in vitro, UVB-irradiated HDF	↓ MMP-1, 3 and ROS production; ↓ procollagen type I reduction; anti-photoaging action via the activation of Nrf2, upregulation of TGF-β, downregulation of AP-1 and MAPK phosphorylation; ↓ UVB-induced VEGF and IL-6, COX-2, iNOS and TNF-α secretion	[65]
*Hylocereus polyrhizus* (Cactaceae)	fruit peel, EE	in vitro, SPF via UV spectroscopy	SPF 35.02	[43]
*Juglans regia* (Juglandaceae)	male flower, ME	in vitro, UVB-irradiated HaCaT	prevents the overexpression of MAPKs, AP-1, MMPs, Smad7; ↓ expression of TIMP-1/2, TGF-β1, Smad3 and procollagen type-1 in cells	[66]
*Kadsura coccinea* (Schisandraceae)	root, stem, leaf and fruit, EE	in vitro, UVA and UVB-irradiated HaCaT	alleviates anti-proliferative and cytotoxic effects of UVA/UVB irradiation on cells; ↓ intracellular ROS level and keratinocyte damage	[67]
*Melaleuca leucadendron* (Myrtaceae)	flower, EE	in vitro, UVB-induced HaCaT	↓ COX-2 expression, ensures protection of DNA damage, prevents the increase in ROS; ↑ levels of the antioxidant enzymes SOD, GPx and CAT	[68]
*Moringa concanensis* (Moringaceae)	stem bark extract	in vitro, SPF via UV spectroscopy, UVA/UVB absorption spectra	SPF 10.46 and broad absorption spectrum (UVA and UVB) ranges	[69]
*Oenanthe javanica* (Apiaceae)	EE	in vivo, UVB-exposed mouse	↑ collagen types I and III productions; ↓ MMP-1 and MMP-3, TNF-α and COX-2 expression	[70]
*Penthorum chinense* (Penthoraceae)	EE	in vitro, HaCaT under UVB or H_2_O_2_ treatment	↑ the promoter activity of the type 1 procollagen gene Col1A1; ↓ MMPs, COX-2, IL-6 expression and HYAL induced by UVB irradiation or H_2_O_2_-induced oxidative stress; ↓ phospho-p38 and phospho-JNK	[71]
*Pradosia mutisii* (Sapotaceae)	ME	in vitro, HaCaT, HDF under UVB or H_2_O_2_ treatment	↓ MMP-1 and MMP-9; ↑ Sirt-1	[72]
*Posoqueria latifolia * (Rubiaceae)	flower, EE	in vitro, SPF via UV spectroscopy	SPF 35, broad-spectrum (UVA-UVB) protection efficacy	[44]
*Ranunculus bulumei * (Ranunculaceae)	aerial part, ME	in vitro, UVB-irradiated HaCaT	↓ mRNA levels of MMP-9, COX-2; ↑mRNA levels of Sirt-1, type-1 procollagen; ↓ phospho-p38; inactivates AP-1	[73]
*Rosa centifolia* (Rosacea)	flower, EE	in vitro, SPF via UV spectroscopy	SPF value of 32, broad-spectrum (UVA-UVB) protection efficacy	[44]
*Rosmarinus officinalis* (Lamiaceae)	leaf, HE	in vivo, UV-irradiated rat	↓ level of GSH, SOD, CAT; ↓ IL-1β, IL-6, and NF-kB; ↓ MMP-1, GM-CSF, NEP	[74]
*Rubus idaeus* (Rosaceae)	EE	in vitro, HaCaT; in vivo, mouse exposed to UVB	alleviate UVB-caused erythema in the skin; ↓ formation of 8-OHdG; recover the expression of Nrf2 and antioxidant enzyme proteins SOD and CAT; ↓ phospho-p38 and NF-κB expression	[75]
*Sideritis raeseri * (Lamiaceae)	aerial part, WEE	in vitro, SPF via UV spectroscopy	SPF 4.54–18.01	[76]
*Sloanea medusula* *Sloanea calva* (Elaeocarpaceae)	leaf, EE	in vitro, SPF via UV spectroscopy	*S. medusula*, SPF 32.5 *S. calva*, SPF 35.4	[42]
*Spatholobus suberectus * (Leguminosa)	stem, WE, EE	in vitro, HaCaT exposed to UVB	↓ ROS production; block MAPK, NF-κB and c-Jun; ↑ Col1a1, ELN, HAS2 expression	[77]
Syzygium formosum (Myrtaceae)	leaf, EE	in vitro, HaCaT exposed to UVB	↓ IL-1 β, IL-6, IL-8 and COX-2 expression	[78]
*Silybum marianum* (Asteraceae)	sylimarin and flavonolignans	in vitro, SPF via UV spectroscopy	absorbs UVB and UVA; SPF 2.01–6.07	[79]
*Washingtonia filifera * (Arecaceae)	seed, EE, WE, ME	in vitro, H_2_O_2_-induced HaCaT; in vitro SPF via UV spectroscopy	↓ ROS generation; SPF 1.52–3.35.	[80]
*Zanthoxylum bungeanum * (Rutaceae)	sanshool, a major component	in vitro, UVB-irradiated HDF; in vivo, mouse	↓ activation of JAK2-STAT3 signaling; ↓ MMP-1 and MMP-3 secretion	[81]

↓, inhibit/suppress/decrease; ↑, enhance/induce/increase; 8-OHdG, 8-hydroxydeoxyguanosine; AP-1, activator protein 1; BE, buthanol extract; Col1a1, collagen type I alpha 1; EE, ethanol extract; ELN, elastin; ERK, extracellular signal-regulated kinase; GM-CSF, granulocyte-macrophage colony-stimulating factor; HaCaT, human keratinocyte cell line; HAS2, hyaluronan synthase 2; HDF, human dermal fibroblast cell line; HE, hexane extract; JNK, c-Jun N-terminal kinase; MAPK, mitogen-activated protein kinases; MCP-3, monocyte chemotactic protein-3; ME, methanol extract; MMP, matrix metalloproteinases; NEP, neprilysin; NF-κB, nuclear factor-kappa B; NIH-3T3, skin fibroblast cells; NHEK, neonatal normal human epidermal keratinocytes; Nrf2, nuclear factor erythroid 2-related factor 2; Sirt-1, sirtuin 1; STAT3, signal transducer and activator of transcription; TIMP, tissue inhibitor of metalloproteinases; WAE, water/aceton extract; WE, water extract; WEE, water/ethanol extract; WME, water/methanol extract.

**Table 2:** The impact of selected plant extracts and natural products of plant origin on skin regeneration and wound healing.

Plant (Family)	Plant Material	Cell/Animal	Effect	Ref
*Agrimonia eupatoria * (Rosaceae)	WE	in vitro, NIH 3T3, HDF and HaCaT; in vivo, rat	↑ ECM deposition, ↑ keratinocyte proliferation/differentiation; ↑ wound TS and contraction rates	[105]
*Angelica polymorpha * (Umbelliferae)	flower absolute	in vitro, HaCaT	↑ cell migration, proliferation and collagen IV synthesis; ↑ phosphorylation of ERK1/2, JNK, MAPK p38 and Akt	[106]
*Annona reticulata* (Annonaceae)	leaf, EE	in vitro, HaCaT	↑ VEGF and Akt; ↑ cell migration and proliferation	[107]
*Astragalus floccosus * (Leguminosae)	root, ME	in vitro, NHDF; in vivo, rat	↑ scratch wound healing, cell proliferation, fibrosis and epithelization	[108]
*Betula pendula* (Betulaceae)	bark, WE	in vitro, HaCaT	strong activities against *S. aureus*, *C. acnes* and *S. epidermidis*; ↑ wound closure	[109]
*Boesenbergia rotunda* (Zingiberaceae)	rhizome, EE	in vitro, HaCaT	↑ ERK1/2 and Akt; ↑ cell migration and proliferation	[110]
*Bursera morelensis* (Burseraceae)	terpenes α-pinene and α-phellandrene	in vivo, mouse	↑ wound contraction due to collagen deposition from the early stages; provided better structure in scar tissue	[111]
*Centella asiatica * (Apiaceae)	WGE	in vitro, HaCaT	positively affected wound healing and cell migration	[112]
*Cumin carvi* (Apiaceae)	seed, WEE	in vivo, rat	healing effects: ↑ total protein content and biomechanical factors; ↑ re-epithelialization, granular tissue, connective tissue, collagen and angiogenesis index; ↓ inflammatory factors	[113]
*Cyclopia* spp. (Fabaceae)	leaf and branch, WE, WEE	in vitro, HaCaT	↑ cell migration	[61]
*Derris scandens* (Fabaceae)	stem, WE, EE	in vitro, HSF	↑ cell migration and wound closure in a scratch assay	[114]
*Digitaria ciliaris * (Poaceae)	flower, EE	in vitro, CCD986sk HaCaT	↑ cell proliferation and migration; ↑ collagen I and IV syntheses; ↑ phosphorylation of ERK1/2 and p38 MAPK	[115]
*Fagus sylvatica* (Fagaceae)	bark, WE	in vitro, HaCaT	strong activities against *S. aureus*, *C. acnes* and *S. epidermidis*; ↑ wound closure	[109]
*Glycyrrihza glabra * (Fabaceae)	root, EE	in vivo, rat	↑ collagen synthesis, ↑ α-SMA, PDGFR-α, FGFR1 and Cytokeratin 14 expression; ↑ angiogenesis and collagen deposition through up-regulation of bFGF, VEGF and TGF-β gene expression levels	[116]
*Garcinia mangostana * (Clusiaceae)	pericarp, EE	in vitro, 3T3-CCL92	↑ fibroblast proliferation and wound recovery	[117]
*Greyia radlkoferi * (Melianthaceae)	leaf, EE	in vitro, HaCaT	antibacterial activity against wound-associated bacteria (*S. aureus*)	[118]
*Hydrangea serrata* (Hydrangeaceae)	leaf, WE	in vitro, HaCaT	improved transcription levels of keratin Ker5, Ker6 and Ker16	[119]
*Jatropha neopauciflora * (Euphorbiaceae)	latex	in vivo, normal and diabetic mouse	accelerated and improved the wound-healing process	[120]
*Nigella sativa* (Ranunculaceae)	seed, EE	in vitro, 3T3-CCL92	↑ cell proliferation and wound recovery	[117]
*Rosmarinus officinalis* (Lamiaceae)	leaf, HE	in vitro, HaCaT	↑ migration and repopulation of keratinocytes at the scratched area and considerably narrowed the scratched gap	[74]
*Salix koreensis* (Salicaceae)	flower absolute	in vitro, HaCaT	↑ cell proliferation, migration and collagen I and IV production; ↑ phosphorylation of Akt, JNK, ERK1/2 and p38 MAPK	[121]
*Sapindus mukorossi* (Sapindaceae)	kernel oil	in vitro, CCD-966SK	↑ cell proliferation and migration; anti-inflammatory and anti-microbial activities; ↑ wound healing, ↓ size of the wound	[122]
*Sorocea guilleminina * (Moraceae)	leaf, WE	in vitro, N3T3; in vivo rat	↑ cell proliferation/migration rate, ↑ wound contraction	[123]
*Ulmus parvifolia* (Ulmaceae)	root bark, ME	in vitro, HaCaT; in vivo, mouse	↑ cell migration; upregulated the expression of the MMP-2 and -9 protein, ↑ TGF-β	[124]
Plant material	Formulation	Cell/animal	Effect	Ref
*Aloe vera*	gel with EE	in vitro, HaCaT, HFF1; in vivo, rat	↑ cell proliferation; promoted wound healing; accelerated re-epithelialization and wound contraction	[125]
*Avicennia schaueriana*	cream with leaf WE	in vivo, mouse	↑ re-epithelialization and the number of fibroblasts, exhibiting a healing activity on skin injuries	[126]
*Caralluma europaea*	ointment with aerial part WEE	in vivo, rat	↑ wound healing	[127]
*Cassia obtusifolia*	gel with aerial part EE	in vivo, rat and mouse	↑ wound healing	[128]
*Clematis simensis*	ointment with leaf WEE	in vivo, mouse and rat	↑ wound contraction and epithelialization; extract reduced inflammation and demonstrated antioxidant activity	[129]
*Cnestis ferruginea*	creams with root bark ME	in vivo, rat	↓ wound size; affected the formation of well-regenerated tissue	[130]
*Convolvulus arvensis*	ointment with stem ME	in vivo, rat	↑ wound closure; improved skin architecture; healing potential comparable to that of gentamycin	[131]
*Centella asiatica*	hydrogel with asiaticoside-rich fraction	in vivo, rabbit	↑ wound healing	[132]
*Cynara humilis*	ointment with root WE and EE	in vivo, rat	↑ wound contraction, epithelialization, ↑ collagen production; ↓ the number of inflammatory cells during wound healing	[133]
*Epilobium angustifolium*	hydrogel with EE, IE and WE	in vitro, HDF	↑ wound healing; activity against *S. pneumoniae*, *E. coli*, *E. faecalis*, *E.* *faecium*, *S. lutea* and *B. pseudomycoides*	[94]
*Ginkgo biloba*	O/W cream with leaf WE	in vivo, diabetic rats	↑ wound closure associated with increased collagen synthesis	[134]
*Loranthus acaci*	gel with aerial part EE	in vivo, rat and mouse	↑ wound healing	[128]
*Marantodes pumilum*	ointment with leaf and root WE	in vivo, rat	↑ wound healing; re-epithelialization, collagen deposition, fibronectin content and fibroblast cells, and fiber transformation from collagen III to I	[135]
*Phlomis russeliana*	gel with aerial part extract	in vivo, mouse	↑ dermal and epidermal regeneration, collagen formation, ↑ TGF-β, VEGF and FGF levels	[136]
*Punica granatum*, *Matricaria chamomilla*	ointment with methanol fraction of pomegranate and chamomile flowers	in vivo, rat	↑ wound healing; activity against *S. aureus*, *S. epidermidis* and *P. aeruginosa* of plant extracts	[137]
*Roylea elegans*	cream with leaf WE	in vivo, rat	↑ wound contraction formation of collagen, and tissue re-epithelialization; ↑ protein, GSH, SOD and CAT levels, ↓ MPO levels; ↑ IL-10, ↓ TNF-α and IL-6	[138]
*Tamarix aphylla*	nanoemulsion W/O with leaf ME	in vivo, rabbit	↑ acid-burn wound-healing process (improved cell attachment at the edge of the wound, collagen content), ↓ healing duration	[139]
*Urtica simensis*	ointment with leaf WME	in vivo, mouse	↑ wound contraction, ↓ periods of epithelialization	[140]
*Virola oleifera*	cream with resin	in vivo, rat	↑ wound contraction; ↓ LPO and protein oxidation	[141]
Plant essential oils	polysaccharide-based hydrogel with eucalyptus, ginger and cumin EO	in vitro, L929 cells; in vivo, mouse	antibacterial activity against *S. aureus* and *E. coli*; ↑ cell migration and improved burn wound healing	[142]
Cinnamaldehyde	nanoemulsion	in vivo, rat	↓ wound size; ↑ CAT and SOD, ↓ NAP3; activity against *S. aureus* and *S. typhimurium*	[143]

↓, inhibit/suppress/decrease; ↑, enhance/induce/increase; ECM, extracellular matrix; EE, ethanol extract; ERK, extracellular signal-regulated kinase; FGF, fibroblast growth factor; HE, hexane extract; IE, isopropanol extract; JNK, c-Jun N-terminal kinase; LPO, lipid peroxidation; MAPK, mitogen-activated protein kinases; ME, methanol extract; MMP, matrix metalloproteinases; MPO, myeloperoxidase; NAP3, cytokine neutrophil-activating protein 3; PDGFR-α, platelet-derived growth factor receptor-α; SMA, smooth muscle actin; TGF-β, transforming growth factor β; TS, tensile strength; VEGF, vascular endothelial growth factor; WE, water extract; WEE, water/ethanol extract; WGE, water/glycerin extract; WME, water/methanol extract.

**Table 3:** Selected plant extracts from various species with anti-aging activity.

Species (Family)	Part/Extract	Method	Results	Ref
*Aegopodium podagraria* (Apiaceae)	WGE	enzyme reaction assay, spectrophotometric method in vitro	↓ ELA and COL activity	[112]
*Aerva lanata* (Amaranthaceae)	EE, WE	enzyme reaction assay, spectrophotometric method in vitro	↓ ELA, COL and HYAL activity	[153]
*Arachis hypogaea* (Fabaceae)	peanut shells, UAE	enzyme reaction assay, spectrophotometric method in vitro	↓ COL activity	[154]
*Artemisia iwayomogi * (Asteraceae)	1% water fraction	in vivo study on 21 women volunteers	anti-wrinkle effect after using O/W cream for 8 weeks; ↓ depth of fine wrinkles on facial skin	[155]
*Asparagus officinalis* (Asparagaceae)	aerial parts, EE	enzyme reaction assay, spectrophotometric method in vitro	↓ MMP-1, ELA and HYAL activity	[156]
*Borago officinalis* (Boraginacea)	aerial parts, ME, WME	enzyme reaction assay, spectrophotometric method in vitro	↓ ELA, COL activity	[157]
*Bruguiera gymnorhiza * (Rhizophoraceae)	leaf, root, twig, fruit, EAE, ME	enzyme reaction assay, spectrophotometric method in vitro	↓ ELA activity	[158]
*Cannabis sativa* (Cannabaceae)	herb, WEE, MAE, UAE	enzyme reaction assay, spectrophotometric method in vitro; application analysis on 15 volunteers in vivo	↓ COL and ELA activity; ↓ TEWL; ↑ skin moisture level	[150]
*Cyclopia* spp. (Fabaceae)	leaf, branches, WE, WEE, WAE, BE	enzyme reaction assay, spectrophotometric method in vitro	↓ COL and HYAL, weak influence on ELA activity	[61]
*Curculigo latifolia* (Hypoxidaceae)	root, steam, leaf, EAE, EE	enzyme reaction assay, spectrophotometric method in vitro	↓ ELA activity	[159]
*Dimocarpus longan* (Sapindaceae)	seed extracts, PET, EAE, EE	enzyme reaction assay, spectrophotometric method in vitro	↓ MMP-1 and HYAL activity	[160]
*Euphorbia characias* (Euphorbiaceae)	leaf, EE	enzyme reaction assay, spectrophotometric method in vitro	↓ ELA, COL and HYAL activity	[64]
*Hydrangea serrata* (Hydrangeaceae)	leaf, WE	in vitro, HaCaT and HDF; clinical study (22 subjects)	↑ skin barrier components and HAS, ↓ mRNA levels of HYAL-1, -2, -3; ↑ mRNA expression of Col1a1; ↑ skin moisture level, ↓ skin wrinkles	[119]
*Nelumbo nucifera* (Nelumbonaceae)	whole flower, stamen, EE	enzyme reaction assay, spectrophotometric method in vitro	↓ ELA, COL and HYAL activity	[161]
Olea europaea (Oleacea)	leaf, WE, PPG, LA; MAE, UAE	enzyme reaction assay, spectrophotometric method in vitro	↓ ELA and COL activity	[162]
*Plectranthus* spp. (Lamiaceae)	aerial part, WE, ME, AE, EAE	enzyme reaction assay, spectrophotometric method in vitro	↓ ELA and COL activity	[163]
*Pradosia mutisii* (Sapotaceae)	ME	in vitro, HaCaT and HDF	↑ expression of moisturizing-related genes HAS-2, TGM-1 and Col1a1 gene	[72]
*Premna odorata* (Verbenaceae)	leaf, EO	enzyme reaction assay, spectrophotometric method in vitro	considerable anti-ELA and anti-HYAL and mild anti-COL potential	[164]
*Rosmarinus officinalis* (Lamiaceae)	leaf, HE	enzyme reaction assay, fluorometric and spectrophotometric methods in vitro	↓ ELA, COL and HYAL activity	[74]
*Spatholobus suberectus* (Fabaceae)	stem, WE, EE	enzyme reaction assay, spectrophotometric method in vitro	↓ ELA activity	[77]
*Thunbergia laurifolia * (Acanthacea)	leaf, EE, SE, RE	enzyme reaction assay, fluorometric and spectrophotometric methods in vitro; in vitro, 3T3 cells	↓ MMP-1, MMP-2, -9 and HYAL activity	[165]
*Vitis vinifera* (Vitaceae)	fruit, WEE	single-blind placebo-controlled in vivo study, 11 volunteers	improvement in skin moisture and elasticity after 12 weeks of applying W/O emulsion	[166]
*Washingtonia filifera * (Arecaceae)	pulp, seed, WE, EE, ME	in vitro, HaCaT	↓ ELA and COL activity	[80]
*Warburgia salutaris* (Canellacea)	bark, WE	enzyme reaction assay, spectrophotometric method in vitro	activity against HYAL > ELA > COL	[167]
*Papaver rhoeas* *Punica granatum* *Clitoria ternatea* *Carthamus tinctorius* *Gomphrena globasa*	flower, WEE	enzyme reaction assay, spectrophotometric method in vitro; application analysis on 15 volunteers in vivo	↓ ELA and COL activity; SPF 20–31; ↓ TEWL; ↑ skin moisture level	[168]
*Cannabis sativa* *Foeniculum vulgare* *Punica granatum* *Vitis vinifera*	seed, EE, WEE, SFE UAE	enzyme reaction assay, spectrophotometric method in vitro	↓ ELA and COL activity	[169]
*Phyllanthus emblica* *Momordica cochinchinensis* *Centella asiatica*	leaf, fruit extract	randomized double-blind placebo-controlled in vivo study, 60 women	significant improvement in skin hydration, elasticity and wrinkles in eye and cheek areas after 60 days of emulsion application containing an extract combination	[151]

**↑**, enhance/increase; ↓, inhibit/decrease; AE, aceton extract; BE, buthanol extract; COL, collagenase; Col1a1, collagen type I alpha 1; EAE, ethyl acetate extract; ELA, elastase; EE, ethanol extract; EO, essential oil; HAS, hyaluronic acid synthase; HE, hexane extract; HYAL, hyaluronidase; LA, lactic acid; MAE, magnetic-stirrer-assisted extraction; ME, methanol extract; MMP, metalloproteinases; PET, petroleum ether; PPG, polypropylene glycol; RE, reflux extraction; SE, Soxhlet extraction; SFE, supercritical fluid extraction; TGM-1, transglutaminase-1; UAE, ultrasound-assisted extraction; WE, water extract; WAE, water/aceton extract; WGE, water/glycerin extract; WEE, water/ethanol extract; WME, water/methanol extract.

**Table 4:** Selected plant species and their anti-tyrosinase properties.

Plant Species	Family	Part of Plant	Ref
*Acanthus mollis*	Acanthaceae	leaves	[192]
*Aerva lanata*	Amaranthaceae	aerial parts	[153]
*Allium galanthum*	Amaryllidaceae	bulbus	[193]
*Allium turkestanicum*			
*Anacamptis pyramidalis*	Orchidaceae	tubers	[194]
*Anacardium occidentale*	Anacardiaceae	leaves	[195]
*Anacardium occidentale*	Anacardiaceae	fruits	[196]
*Andropogon virginicus*	Poaceae	aerial parts	[197]
*Angelica keiskei*	Umbelliferae	leaves, roots	[198]
*Arachis hypogaea*	Fabaceae	peanut shell	[154]
*Areca catechu*	Palmaceae	fruits	[195]
*Arctium minus*	Asteraceae	flower heads, leaves, roots	[199]
*Artemisia verlotiorum*	Asteraceae	whole plant	[200]
*Atractylodis macrocephalae*	Asteraceae	rhizomes	[201]
*Berberis thunbergii*	Berberidaceae	leaves	[202]
*Bergenia pacumbis*	Saxifragaceae	plant and its rhizomes	[203]
*Blepharis linariifolia*	Acanthaceae	aerial parts	[204]
*Bletilla striata*	Orchidaceae	tubers, fibrous roots	[205]
*Breynia retusa*	Phyllanthaceae	leaves	[206]
*Bridelia ferruginea*	Phyllanthaceae	leaves, stem bark	[207]
*Bruguiera gymnorhiza*	Rhizophoraceae	leaves, roots, fruits	[158]
*Cakile maritima*	Brassicaceae	fruits, leaves, stems	[208]
*Cannabis sativa*	Cannabaceae	seeds	[169]
*Carthamus tinctorius*	Asteraceae	seeds	[209]
*Celastrus hindsii*	Celastracea	leaves	[210]
*Cercis glabra*	Fabaceae	leaves	[211]
*Cladium mariscus*	Cyperaceae	seeds	[212]
*Clausena indica*	Rutaceae	roots	[213]
*Combretum micranthum*	Combretaceae	leaves	[196]
*Crotalaria burhia*	Fabaceae	aerial parts, roots	[214]
*Croton hirtus*	Euphorbiaceae	aerial parts	[215]
*Cudrania tricuspidata*	Moraceae	fruits	[216]
*Cytinus hypocistis*	Cytinaceae	aerial parts	[217]
*Dianella ensifolia*	Liliaceae	roots	[218]
*Dodonaea viscosa*	Sapindaceae	stems	[219]
*Elaeagnus angustifolia*	Elaeagnaceae	fruits, leaves	[220]
*Euphorbia hirta*	Euphorbiaceae	whole plant	[196]
*Feijoa sellowiana*	Myrtaceae	leaves	[221]
*Foeniculum vulgare*	Apiaceae	seeds	[169]
*Glochidion zeylanicum*	Phyllanthaceae	leaves	[195]
*Girardinia diversifolia*	Urticaceae	shoot tips	[222]
*Helichrysum rutilans*	Asteraceae	aerial parts	[223]
*Heliotropium procumbens*	Boraginaceae	aerial parts	[224]
*Heliotropium crispum*	Boraginaceae	whole plant	[225]
*Hibiscus tiliaceus*	Malvaceae	leaves	[226]
*Hypericum montbretii*	Hypericaceae	aerial parts	[227]
*Hypericum origanifolium*			
*Iris pseudacorus*	Iridaceae	aerial parts, rhizomes	[228]
*Jatropha curcas*	Euphorbiaceae	stems, bark, leaves	[229]
*Jatropha gossipiifolia*			
*Limonium effusum*	Plumbaginaceae	aerial parts	[230]
*Limonium sinuatum*			
*Litchi chinensis*	Sapindaceae	roots	[231]
*Lonicera japonica*	Caprifoliaceae	whole plant	[232]
*Mangifera caloneura*	Anacardiaceae	leaves	[195]
*Manilkara kauki*	Sapotaceae	fruits, leaves, seeds, stem bark, woods	[233]
*Matthiola incana*	Brassicacea	leaves, flower buds	[234]
*Melastoma normale*	Melastomacea	roots	[235]
*Momordica cochinchinensis*	Cucurbitacea	fruits (pulp, aril, seed)	[236]
*Monotheca buxifolia*	Sapotaceae	leaves, stems	[237]
*Nelumbo nucifera*	Nelumbonaceae	whole flower, stamen	[161]
*Onosma bourgaei,*	Boraginaceae	aerial parts	[238]
*Onosma trachytricha*			
*Paliurus spina-christi*	Rhamnaceae	fruits, leaves, stems	[239]
*Pistacia lentiscus*	Anacardiaceae	leaves	[240]
*Phaseolus vulgari*	Fabaceae	seed coat	[241]
*Phytolacca dioica*	Phytolaccacea	fruits	[242]
*Plectranthus ecklonii*, *P. namaensis*, *P. zuluensis*	Lamiacea	aerial parts	[243]
*Punica granatum*	Punicaceae	seeds	[169]
*Rheum palmatum*	Polygonacea	roots, rhizomes	[244]
*Rhizophora racemosa*	Rhizophoraceae	leaves, stem bark	[245]
*Rhizophora apiculata*	Rhizophoraceae	leaves	[226]
*Rhizophora mucronata*			
*Rosa platyacantha*	Rosaceae	flowers, leaves, buds	[246]
*Rubus fraxinifolius*	Rosaceae	leaves	[247]
*Salvia chamelaeagnea,*	Lamiacea	aerial parts	[243]
*Salvia dolomitica*			
*Sartoria hedysaroides*	Fabaceae	aerial parts	[248]
*Schisandra chinensis*	Schisandraceae	fruits	[249]
*Secamone afzelii*	Asclepiadaceae	leaves	[250]
*Streblus taxoides*	Moraceae	wood	[251]
*Strobilanthes glutinosus*	Acanthaceae	whole plant	[252]
*Tambourissa peltat*	Monimiaceae	fruits, flowers, leaves	[200]
*Vitis amurensis*	Vitaceae	root	[253]
*Vitis vinifera*	Vitaceae	seeds	[169]
*Warburgia salutaris*	Canellacea	barks	[167]
*Zingiber kerrii*	Zingiberaceae	rhizomes	[254]
*Ziziphora taurica*	Lamiaceae	aerial parts	[255]

The in vitro spectrophotometric enzyme tyrosinase inhibition assay was used to measure anti-tyrosinase activity, compared with kojic acid or β-arbutin as a reference tyrosinase inhibitor.

**Table 5:** Selected plants with identified essential oil compounds and a description of their aroma.

Family	Aromatic Plants	Extraction of EO	Single Constituent	Aroma Description	Ref.
Lamiaceae	*Lavandula* *officinalis*	hydrodistillation of air-dried flowers	linalool, linalyl acetate, geraniol, β-caryophyllene, lavandulyl acetate	fresh, herbaceous, floral	[261]
	*Origanum* *vulgare*	hydrodistillation of air-dried aerial parts	carvacrol, γ-terpinene, *p*-cymene, *trans*-sabinene hydrate, thymol	warm, spicy, camphoraceous	[262]
	*Thymus* *vulgaris*	hydrodistillation of shade-dried flowers and leaves	thymol, γ-terpinene, *p*-cymene, linalool, myrcene, α-pinene, α-thujene	strong, spicy, herbaceous	[263]
	*Mentha* *piperita*	hydrodistillation of shade-dried aerial parts	camphane, menthone, menthol, *β*-pinene, pulegone, *β*-cubebene, α-pinene, γ-terpinene, γ-carane, piperiton	fresh, sweetish, menthol	[264]
	*Hyssopus* *officinalis*	steam distillation, simple hydrodistillation and hydrodistillation in Dean–Stark apparatus of air-dried flowering aerial parts	elemol, spathulenol, α-eudesmol, γ-eudesmol, virdiflorol, hedycaryol, isopinocamphone, cis-jasmone.	fresh, herbal, slightly sweet, camphorous	[265]
Apiaceae	*Pimpinella* *anisum*	hydrodistillationof mature fruits	*trans*-anethole, *γ*-himachalene, *trans*-pseudoisoeugenyl 2-methylbutyrate, *cis*-dihydrocarvone, methyl chavicol, *α*-himachalen, *β*-himachalene	fresh, warm, sweet, mildly pungent	[266]
	*Carum* *carvi*	hydrodistillation and microwave-assisted hydrodistillation of air-dried seeds	carvone, limonene, apiole, andrographolide, aromadendrene, β-cadinene, friedelanol, barrigenol, 3-benzyloxyphenol	pungent, anise-like, herbaceous	[267]
Rutaceae	*Citrus* *limon*	hydrodistillation of peels	limonene, α-citral, β-pinene, α-terpinene, β-elemene, neryl acetate	sharp, lemon, sweet	[268]
	*Citrus* *paradisi*	molecular distillation from cold-pressed fruits	limonene, β-myrcene, α-pinene, sabinene (0.60%), carvone (0.41%), cis-limonene oxide (0.43%), and trans-limonene oxide (0.33%), caryophyllene (0.20%), β-cubebene (0.14%), α-copaene (0.13%),	fresh, sharp, citrus	[269]
Verbenaceae	*Verbena* *officinalis*	steam distillation of leaves	limonene, 1,8-cineole, ar-curcumeme, caryophyllene oxide, spathulenol	lemony scent with sweet, fruity undertones	[270]
Lauraceae	*Cinnamomum verum*	hydrodistillation of shade-dried leaves	eugenol, linalol, benzyl benzoate,	sweet, spicy, slightly woody, clove-like	[271]
Asteraceae	*Anthemis* *nobilis*	hydrodistillation of shade-dried flowers	en-yn-dicycloether, β-caryophyllene, aristolene epoxide, germacrene D, widdrol, cis-caryophyllene	crisp, sweet, herbal, floral, soft fruity (reminiscent of apples)	[272]
Myrtaceae	*Eucalyptus* *globulus*	steam distillation of dried leaves	eucalyptol, α-pinene, p-cymene, β- myrcene, terpinen-4-ol, γ-terpinene	fresh, camphoraceous, medicinal	[273]
	*Melaleuca* *alternifolia*	steam distillation of young branches and leaves	terpinen-4-ol,-terpinene, 1,8-cineole, p-cymene	fresh, camphoraceous	[274]
	*Syzygium* *aromaticum*	supercritical fluid extraction assisted by cold pressing buds	eugenol, eugenyl acetate, β- caryophyllene, α-humulene	clove, strong	[275]
Geraniaceae	*Pelargonium* *graveolens*	hydrodistillation of fully grown aerial parts	citronellol, geraniol, caryophyllene oxide, menthone, linalool, β-bourbonene, *iso*-menthone, geranyl formate	floral, sweet, rose-like with minty undertones	[276]

## Data Availability

Not applicable.
